# A case control association study of *COMT* gene polymorphism (I/D) with type 2 diabetes and its related factors in Pakistani Punjabi population

**DOI:** 10.1186/s40200-015-0166-x

**Published:** 2015-05-06

**Authors:** Maryam Zain, Fazli Rabbi Awan, Sidra Amir, Shahid Mahmood Baig

**Affiliations:** Diabetes and Cardio-Metabolic Disorder (D&C-MD) Laboratory, Health Biotechnology Division, National Institute for Biotechnology and Genetic Engineering (NIBGE), Jhang Road, P.O. Box.577, Faisalabad, Pakistan; Pakistan Institute of Engineering and Applied Sciences (PIEAS), Islamabad, Pakistan

**Keywords:** Type 2 diabetes, Family history, Nephropathy, Pakistani

## Abstract

**Background:**

The *Catechol-O-Methyl Transferase* (*COMT)* gene polymorphism (I/D of C nucleotide at base position 900) has been previously implicated in the development of type 2 diabetes (T2D) and kidney disease. So, aim of this study was to find association of I/D polymorphism with T2D, and its associated factors like family history and nephropathy (End Stage Renal Disease, ESRD) patients in a cohort of Pakistani Punjabis.

**Methods:**

Genomic DNA was extracted from human subjects divided as four study groups: controls (n = 46), diabetics (n = 46), diabetics with nephropathy/ESRD (n = 53), and non-diabetics without nephropathy/ESRD (n = 43). The 900 I/D C polymorphism in the *COMT* gene was tested by PCR-RFLP method. Genotype and allele frequencies as well as Odds Ratios were calculated for these groups. Groups were compared statistically for the analysis of genotypes, alleles, biochemical parameters as well as disease status.

**Results:**

In comparison with control group (non-diabetic, non-nephropathy), there was no significant difference in rest of the three groups for allele or genotype frequencies of *COMT* gene. However, Chi square (χ^2^) analysis identified a significant (p = 0.02) correlation of the 900 I/D C polymorphism with family history of diabetes, as it was found that greater number (74%) of patients having I allele had a positive family history of T2D.

**Conclusions:**

A significant correlation of the *COMT* polymorphism (900 I/D C) with the family history of T2D has been observed, which has not been previously reported in Pakistani Punjabi population, however, this preliminary finding requires further validation studies.

## Introduction

Diabetes mellitus (DM) is a metabolic disorder caused by the chronically high blood glucose levels in the body, which may be the consequence of defect in insulin secretion, insulin action or combination of both [1]. The type 2 diabetes (T2D) which is the predominant form of diabetes in adults leads to various complications of vital organs and tissues. One such complication is the Diabetic Nephropathy (DN), a kidney disease due to hyperglycemia that affects about 25-40% of the diabetics. According to the Centers for Disease Control (CDC, USA), kidney disease is the ninth leading cause of death [2] and DN is the main cause of the end stage renal disease (ESRD) [2]. Diabetes brings unique pathological changes in the kidney structure and the progression of the kidney disease is accelerated if diabetes is accompanied with hypertension [3]. Several pathophysiological pathways have been implicated in the development and progression of diabetic nephropathy, and one of these is the dopaminergenic pathway. Dopamine system is an important modulator of the renal differentiation, and is very important for the regulation of salt metabolism. Dopamine acts as natriuretic hormone by the inhibition of the Na^+^- K^+^-ATPase and other sodium transporters in several tubular segments. The *Catechol-O-Methyl Transferase* (*COMT)* is one of the important genes of the dopaminergenic pathway with a main function of COMT is to remove toxic metabolites from the body. Hence, *COMT* gene has been studied biologically as it codes for two protein variants which differ from each other on the basis of being soluble and membrane bounded known as Soluble Catechol-O-Methyl Transferase (S-COMT) and Membrane-bound Catechol-O-Methyl Transferase (MB-COMT). Owing to importance of COMT, it is important to study the function and genetics of COMT in humans to devise targeted therapies for patients with diabetes and kidney related problems.

A genome – wide linkage scan for genes controlling urinary albumin excretion revealed the linkage of chromosome 22 with diabetic nephropathy [4]. Later research confirmed that the *COMT* gene is located on the long (q) arm of chromosome 22 at position 11.21. One of the insertion/deletion (Ins/Del) single nucleotide polymorphism of C nucleotide in this gene at base position 900 (i.e. 900 I/D C) has been positively correlated with diabetic kidney disease [5]. The COMT is located in the proximal tubular epithelial cells of the kidney where the dopamine synthesis takes place. According to a study conducted in the Chinese population, it has been reported that dopaminergic pathway plays a potential role in the sodium retention in peripheral tubules and also in regulating blood pressure [6]. An association of dopaminergenic pathway genes with kidney diseases among type 2 diabetics was investigated in Asian Indian population, and the results showed positive correlation of *COMT* gene polymorphism (900 I/D C) with chronic renal insufficiency [5]. Some experimental evidences indicate that the administration of nitecapone, which is an inhibitor of dopamine metabolizing enzyme, COMT (EC 2.1.1.6) has been found to effectively abolish glomerular hypertension, and reduce progression to glomerulosclerosis by inhibiting Na^+^-K^+^-ATPase activity [7,8].

Thus, due to the significant role of the *COMT* gene polymorphism in kidney functions and diabetes, in this study, we investigated the associations of this risk polymorphism (900 I/D C) in *COMT* gene by PCR-RFLP analysis through case control association study in control and diabetic patients with or without kidney disease in Pakistani Punjabi population.

## Material and methods

A total of 191 subjects divided in four groups were included in this study. The groups were: **G1-C** (Healthy Controls no diabetes and no nephropathy, n = 46, age (mean ± SD years) and percentage of males = 45.6 ± 8.4 (48%), females = 46.2 ± 8.4 (52%)), **G2-D** (Type 2 diabetics but without nephropathy, n = 46, age (mean ± SD years) and percentage of males = 79.8 ± 10.4 (64%), females = 54.3 ± 10.4 (36%)), **G3-DN** (Type 2 Diabetics with Nephropathy / ESRD, DN: n = 53, age (mean ± SD years) and percentage of males = 54.2 ± 9.7 (57%), females = 55 ± 6.7 (43%)), **G4-N** (Non-diabetics but having Nephropathy/ESRD called as Control Nephropathy/Nephropathy, n = 43, age (mean ± SD years) and percentage of males = 47.7 ± 11.7 (56%), females =47.8 ± 13.2 (44%)). Samples from **G1-C** and **G2-D** were either collected by arranging diabetes camps (locally called sugar camps) in schools, public places or through personal contacts at Faisalabad District, Pakistan, while samples from **G3-DN** and **G4-N** were collected from the Dialysis Units of Allied Hospital and District Head Quarters Hospital, Faisalabad, Pakistan. All subjects were above 35 years of age, the healthy control group (**G1-C**) inclusion parameters were: no history of diabetes in their families, their consent to participate in the study, no hypertension, no kidney disease, no cancer, and no severe infectious disease. The diagnosis of diabetes was made according to American Diabetes Association criteria. The individuals having systolic and diastolic blood pressures above 120/80 mmHg were considered as hypertensive. After explaining the aims of the study, a written informed consent was obtained from each subject, and the study was approved by the institutional (National Institute for Biotechnology and Genetic Engineering, Faisalabad, Pakistan) ethics review committee.

### Clinical characteristics of subjects

Various demographic and anthropometric measurements were taken along with blood samples from the subjects for DNA extraction and for biochemical analysis. Clinically important analytes such as hemoglobin (Hb), blood glucose, urea, creatinine, cholesterol, and triglycerides were measured on a semi-automated clinical chemistry analyzer (Microlab 300, Merck) (Results not presented here).

### Genotyping

The genotyping for *COMT* gene I/D polymorphism (900 Ins/Del C) was carried out by PCR coupled with Restriction Fragment Length Polymorphism (RFLP). Three different genotypes were observed II, DD and ID for *COMT* gene polymorphism (I/D). The already reported primers [5] were used for carrying out the PCR for amplification of *COMT* gene. The primers used for *COMT* gene polymorphism (I/D) were F-COMT: 5′GACAACGTGATCTGCCCAGG while melting temperature (Tm) was 64.5 °C and R-COMT: 5’GAGGTGTGCTTTGCATTTAG with Tm 58.4 °C. PCR for *COMT* amplification was performed in 0.2 ml tubes containing 30 μL reaction mixture in total. The reaction mixture was prepared by adding 2.0 μL samples DNA dilution, 3 μL 10X PCR buffer (750 mM Tris-HCL, pH 8.8 at 25°C), 1.8 μL MgCl_2_, 1.8 μL dNTPs, 0.6 μL of each forward and reverse primer and 0.3 μL Taq DNA polymerase (Fermentas, EU) and 19.9 μL water to make up the volume 30 μL. The reaction mixture was taken through thermocycling condition consisting: 10 minutes of denaturation at 94°C for template denaturation followed by 35 cycles of amplification each consisting of 3 steps; one minute at 94°C; one minute at 59°C; and one minute at 72°C for final extension. Final 10 minutes at 72°C for Taq DNA polymerase to synthesize any remaining un-extended strands. The PCR product of *COMT* gene was 279 bp which has one restriction site for the 6 cutter *BglI* restriction enzyme. The restriction reaction using the fast digest *Bgl* I (Fermentas) was performed in 1.5 mL eppendorf tubes containing 30 μL total reaction mixture. The reaction mixture was digested at 37°C for 1 hour and run on 2.5-3% agarose gel.

### Statistical analysis

All the statistical analysis was performed by using SYSTAT 11 for windows software (version 11, Systat Software Inc., Chicago, Illinois, USA) and Graphpad Instat (version 3.50). Clinical characteristics of the subjects were given as Mean ± SD. The p-value for calculating the significance of the difference among the clinical characteristics of different groups was checked by the one-way ANOVA (ANalysis Of Variance), while disease outcome was studied by using logistic regression analysis and results presented as OR (95% Confidence Interval). All the genotypes were tested for Hardy-Weinberg equilibrium. Chi square (χ^2^) analysis was performed for disease association in groups.

## Results

**Figure 1:**
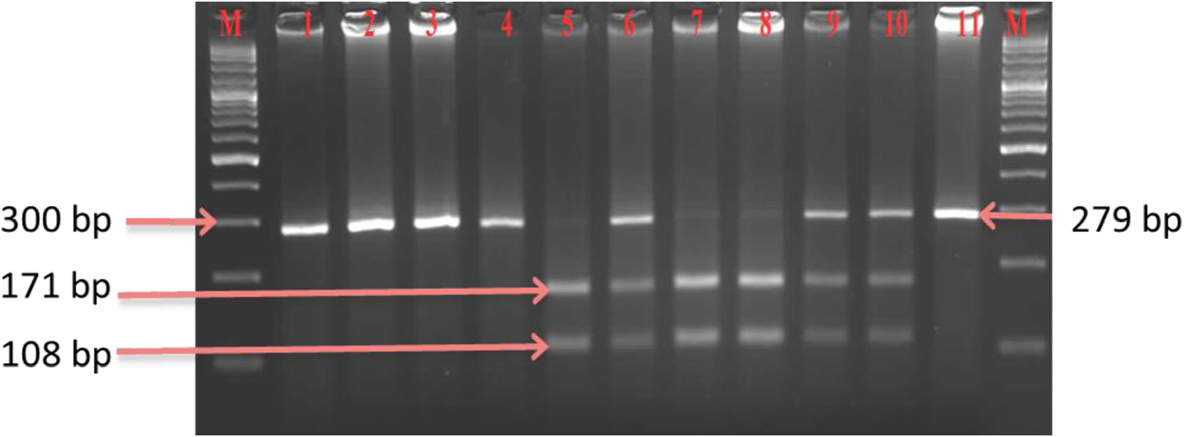
PCR-RFLP based genotypes of *COMT* gene polymorphism (I/D). Lanes M on both ends contain the 100 bp DNA ladder, Lane 1 contains the PCR product without the restriction enzyme, Lanes 2,3,4 and 11 contains the II genotype, Lane 5, 7, and 8 contains DD genotype and Lanes 6, 9 and 10 contain the ID genotype on 2.5% agarose gel.

*COMT* genotypes (II, ID, DD) were, firstly amplified by PCR by subjecting the genomic DNA of all the subjects under study and then amplicons were separately subjected to restriction enzyme based RFLP assay. Figure 1 shows the PCR amplicon of 279 bp size of the target region of *COMT* gene, which was subjected for the identification of *COMT* genotypes (II, ID or DD). Therefore, these PCR products were further restricted by a restriction enzyme to elucidate the genotypes, and the results are shown in Figure 1 with II (279 bp), DD (171 bp and 108 bp) and ID (279 bp 171 bp and 108 bp) genotypes in some of the representative samples from the studied subjects.

Baseline characteristics of all four groups were tested for the association with *COMT* gene polymorphism. There were no significant differences among the four groups for all of the parameters as Body Mass Index (BMI), blood pressure, hemoglobin, blood glucose, urea, creatinine, cholesterol, and triglycerides. All the results were not significant with reference to the genotypes (results not presented here).

The genotype and allele frequencies for *COMT* gene polymorphism were in accordance with Hardy-Weinberg equilibrium. The allele frequency for I was 0.40 and 0.60 for D allele. The genotype frequency for II was 29 (15.4%), for ID was 96 (50.3%) and for DD was 66 (34.3%) in the study subjects. The Table 1 shows allele and genotype frequencies of *COMT* polymorphism (I/D) for each group individually. This table (Table 1) also shows the genotype and allelic distribution of I/D polymorphism for *COMT* gene between the cases and controls. There was no significant difference between the genotype and allele frequency among the three groups (**G2-D**, **G3-DN** and **G4-N**) versus controls (**G1-C)**.

**Table 1:** **Allele and genotype frequency of** ***COMT*** **polymorphism (I/D) of study cohorts**

	**G1-C**	**G2-D**	**G3-DN**	**G4-CN**
**Genotypes**				
Genotyped numbers*	*46*	*46*	*56*	*43*
**n (%)**				
II	7 (15.2)	8 (17.3)	8 (14.2)	4 (9.3)
ID	23 (50.0)	23 (50.0)	31 (55.3)	20 (46.5)
DD	16 (34.7)	15 (32.6)	17 (30.3)	19(44.1)
p value**	***-***	***0.95***	***0.85***	***0.55***
Allele Frequency I	37 (40.2)	39 (42.3)	28 (32.5)	28 (32.5)
D	55 (59.7)	53 (57.6)	58 (67.4)	58 (67.4)
p value**	***-***	***0.76***	***0.80***	***0.81***
OR (95% CI)	-	1.09 (0.60-1.96)	1.07 (0.61-1.88)	0.56 (0.29-1.07)

*G: Group, C: Control, D: Type 2 Diabetics: DN: Diabetic Nephropathy, CN: Non-diabetic nephropathy, OR: Odds Ratio, 95% CI: 95% Confidence Interval. **The comparisons of the p value are among Control group (G1-C) with other three groups (G2-D, G3-DN, G4-CN) for genotype and allele distributions. The Odds Ratio is calculated by logistic regression analysis.

The Chi square analysis for the association of *COMT* polymorphism (I/D) with family history of diabetes, nephropathy and hypertension was also performed (Table 2). An important though preliminary novel finding from this analysis was a significant correlation of the *COMT* gene polymorphism with the family history of disease. No such correlation has been previously reported for this gene at least in Pakistani population. From the Chi-square analysis (Table 2) for *COMT* polymorphism, it is evident that the high percentage (74%) of subjects with family history of diabetes had II genotype. The percentage of the people with the ID and DD genotype having hypertension was also higher but the p-value showed that the result was not significant (p = 0.93). From the Chi-square analysis for nephropathy the results were also not significant as the p-value was more than 0.05.

**Table 2:** **Effect of** ***COMT*** **gene polymorphism (I/D) on family history of diabetes, hypertension and nephropathy**

**Status**		**II**	**ID**	**DD**	*p value
**Family history of diabetes**	Yes	20 (74.0%)	45(48.3%)	41 (64.1%)	0.02
	No	7 (25.9%)	48(51.6%)	23 (35.9%)	
**Hypertension	Yes	10 (37.0%)	40 (44.0%)	35 (53.0%)	0.31
	No	17 (62.9%)	51(56.0%)	31 (46.9%)	
***Nephropathy	Yes	12 (44.4%)	51 (52.6%)	36 (53.7%)	0.70
	No	15 (55.6%)	46 (47.4%)	31 (46.2%)	

*p value is considered as significant when p <0.05. **Hypertension: when systolic and diastolic blood pressure was more than 120/80 mm Hg. ***Nephropathy: when patients have end stage renal disease and were on dialysis. Comparison for yes/no in the same category of status was calculated by the chi square analysis.

## Discussions

The catechol-o-methyl transferase (*COMT)* gene is an important member of the dopaminergenic pathway. This pathway has been investigated in some studies for its association with diabetes and kidney disease. Hence, the present report describes a case-control association study of insertion/deletion polymorphism of C nucleotide at base position 900 in the *COMT* gene (i.e. 900 I/D C) for its association with diabetes and nephropathy along with related parameters e.g. hypertension and family history of disease.

The *COMT* was chosen for the present investigation as it has been previously reported to be involved in the regulation of blood pressure through catecholamine metabolism. Moreover, dopamine owing to its potential role as natriuretic hormone is implicated to play a role in the development and function of the kidney. Such role of dopamine has been supported from the functional studies using the COMT inhibitor in the diabetic kidney disease in rats [7,8]. Genetic association studies of the dopamine receptor genes and *COMT* were linked to essential hypertension [9].

Furthermore, dopamine also regulates the activity of Angiotensin II which is the key component of Renin Angiotensin Aldosterone System (RAAS) pathway. So, an interplay of dopamine and Angiotensin II could disturb the vascular tone, sodium ion balance and renal injury [10]. Experiments in rats have shown that COMT inhibition could reverse renal abnormalities, and thus can provide protection from the development of diabetic nephropathy [8].

Association of *COMT* gene with diabetes and nephropathy has been reported in a study conducted in the Asian Indian population in which a genetic variant showed association for diabetic nephropathy [5]. Owing to its importance in metabolic disorders, the *COMT* was selected as candidate gene for polymorphism of genotypes in a small group of Pakistani Punjabi population from Faisalabad region. As the population of this region was shared between the two countries (India and Pakistan) prior to independence and both regions have similar environmental conditions. Additionally, the association of common biochemical and clinical parameters was assessed for this *COMT* polymorphism with diabetes and nephropathy in Pakistani Punjabi population.

## Conclusions

The main finding of this study was a positive correlation of family history of diabetes with *COMT* (900 I/D C) polymorphism, which has not been previously reported. Since, this is a preliminary finding from a small number of samples; it is therefore, suggested that a large number of samples should be analyzed in future to validate this result. If such an association is established, it will help to minimize the risk of development of disease in genetically predisposed individuals by earlier interventions.

## Abbreviations

COMT: Catechol-O-Methyl Transferase
T2D: Type 2 Diabetes
ESRD: End Stage Renal Disease
PCR-RFLP: Polymerase Chain Reaction-Restriction Fragment Length Polymorphism
ADA: American Diabetes Association
DM: Diabetes mellitus
DN: Diabetic nephropathy
CDC: Centers for disease control
MB-COMT: Membrane-Bound Catechol-O-Methyl Transferase
G1-C: Group 1-Control
G2D: Group 2- Diabetic
G3-DN: Group 3- Diabetic Nephropathy
G4-N: Group 4- Nephropathy
Hb: Haemoglobin
Tm: Melting temperature
ANOVA: Analysis of variance
BMI: Body Mass Index
